# Beyond Correlation: Establishing Causality in Protein Corona Formation for Nanomedicine

**DOI:** 10.1021/acs.molpharmaceut.5c00262

**Published:** 2025-04-09

**Authors:** Arshia Rafieioskouei, Kenneth Rogale, Amir Ata Saei, Morteza Mahmoudi, Borzoo Bonakdarpour

**Affiliations:** † Department of Computer Science and Engineering, Michigan State University, East Lansing, Michigan 48823, United States; ‡ Center for Translational Microbiome Research, Department of Microbiology, Tumor and Cell Biology, Karolinska Institutet, Stockholm 17165, Sweden; ¶ Department of Radiology and Precision Health Program, Michigan State University, East Lansing, Michigan 48823, United States

**Keywords:** actual causality, correlation, protein corona, proteomics, small molecules

## Abstract

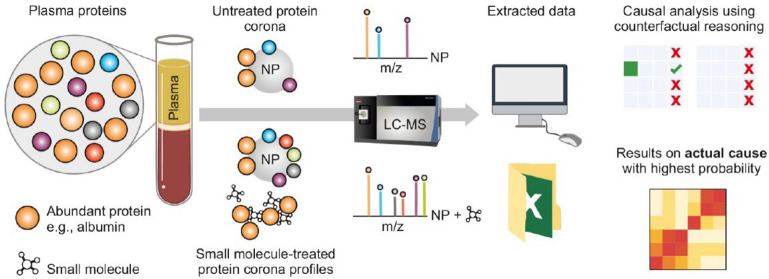

In contemporary studies on the role of the protein corona in specific biological applications, identifying *correlation* is widely used to draw conclusions from observations and statistical methods, yet it merely identifies associations without establishing a direct influence between variables. This over reliance on observation can lead to spurious connections where co-occurrence does not imply causation. In contrast, a *causality*-focused approach asserts the direct impact of one variable on another, offering a more robust framework for inference and the drawing of scientific conclusions. This approach allows researchers to better predict how changes in a nanoparticle’s physicochemical properties or biological conditions will affect protein corona composition and decoration, in turn affecting their safety and therapeutic/diagnostic efficacies. As a proof of concept, we explore the concept of “actual causality” (introduced by Halpern and Pearl) to mathematically prove how spiking small molecules, including metabolites, lipids, vitamins, and nutrients, into plasma can induce diverse protein corona patterns on identical nanoparticles. This approach significantly enhances the depth of plasma proteome profiling. Our findings reveal that among the various spiked small molecules, phosphatidylcholine was the actual cause of the observed increase in the proteomic depth of the plasma sample. By considering the concept of causality in the field of protein coronas, the nanomedicine community can substantially improve the ability to design safer and more efficient nanoparticles for both diagnostic and therapeutic purposes.

## Introduction

Almost all interpretations in the literature regarding the protein corona rely on *correlation* analysis,^1−6^ as opposed to identifying the *causal* reason behind our scientific observations. Causal analysis seeks to identify the cause of an event, while correlation shows a statistical relationship based solely on observations. Causality is a way of describing the logical dependence of events on one another. It is well-known that correlation does not imply causation. For example, during summer, both shark attacks and ice cream sales increase. While there is a correlation, there is no causation; the actual cause is the summer season itself, which drives both activities. One might mistakenly think that eating ice cream causes shark attacks, but the actual cause of both is the higher summer temperatures, which lead to both increased ice cream consumption and more people swimming in the ocean.

**Figure 1:**
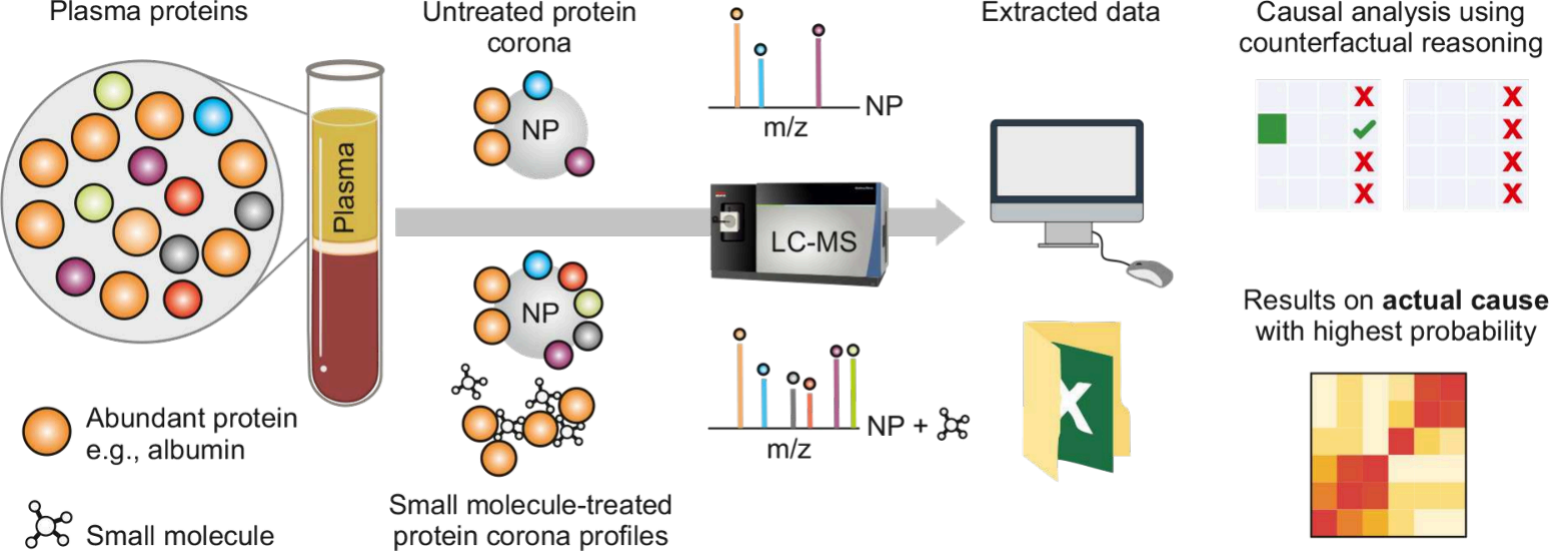
Scheme illustrating the overview of our work. The introduction of small molecules to plasma can modify the interaction sites of proteins with nanoparticles, resulting in diverse corona profiles. This occurs because each small molecule initially interacts with plasma proteins, thereby altering their affinities for the nanoparticle surface. By analyzing the proteomic outcomes of identical nanoparticles after their interactions with plasma treated with various small molecules, we observe a significant improvement in proteome coverage compared to using untreated plasma. The selection of small molecules plays a crucial role in enhancing plasma coverage through the protein corona. Therefore, a thorough causal analysis of the protein corona composition is essential to identify which small molecules are primarily responsible for increasing the number of quantified plasma proteins within the corona.

This work introduces an innovative technique to conduct causal inference on how changes in a nanoparticle’s physicochemical properties or biological conditions will affect protein corona composition and decoration, in turn affecting its safety and therapeutic/diagnostic efficacy. Our idea is to employ the mathematically rigorous concept of *actual causality*,^7^ which does not rely on statistical correlations (see Figure 1 for details).

As a proof of concept, we showcase the significance of our method by focusing on the protein corona: a layer of evolving biomolecules, primarily proteins, that forms on the surface of nanoparticles when they interact with biological fluids, including human plasma.^1,8^ This corona imparts a new biological identity to the nanoparticles, which becomes the point of contact with biosystems, including cells. The type, amount, and conformation of proteins on the nanoparticle surface determine how biosystems perceive and respond to the nanoparticles.^9,10^ Therefore, the protein corona plays a critical role in dictating nanoparticle interactions with biosystems. Recent advances in the field have revealed that the biological identity of identical nanoparticles can vary significantly when exposed to plasma proteins from individuals with diverse health profiles.^11,12^ This suggests that the biological fate and pharmacokinetics of even identical nanoparticles could differ substantially depending on the individual’s unique plasma protein spectrum. Beyond influencing pharmacokinetics and biological fate, the protein corona formed around nanoparticles holds unique potential for disease diagnosis through biomarker discovery.^13^ Specifically, the protein corona reduces plasma protein complexity, thereby improving the detection capacity for low-abundance proteins.^14,15^ This is due to the protein corona’s unique ability to deplete highly abundant plasma proteins and enrich low-abundance ones on the nanoparticle surface.

Several methods have been developed to enhance the capacity of the protein corona for capturing a higher number of low-abundance proteins.^1^ One effective strategy involves utilizing multiple nanoparticles with diverse physicochemical properties.^14,15^ Each additional nanoparticle can capture different plasma proteins, and by combination of all employed nanoparticles, the overall detection of plasma proteins can be significantly enhanced. Another effective strategy involves spiking small molecules into plasma prior to incubation with nanoparticles.^16^ Small molecules interact with plasma proteins, potentially hindering their attachment to the nanoparticle surface or altering their binding sites, thereby modifying the composition of the protein corona.^16,17^ By implementing this strategy, we observed a significant increase in the number of proteins attached to nanoparticles.^16^ For instance, using protein coronas from polystyrene nanoparticles exposed to plasma spiked with small molecules (e.g., glucose, triglyceride, diglycerol, phosphatidylcholine, phosphatidylethanolamine, l-α-phosphatidylinositol, inosine 5-monophosphate, and B complex vitamins together with their combinations titled “sauce 1 and sauce 2”), we achieved a 2.63-fold increase in the number of quantified plasma proteins compared to the protein corona without spiked small molecules.^16^

In this work, using mass spectrometry data from our small molecule protein corona study,^16^ we investigate the underlying causes of the increased number of quantified plasma proteins through an actual causality approach. Specifically, we aim to identify which spiked small molecules contributed to the observed increase and to what extent. Our findings could pave the way for a new dimension in protein corona research, where the causal relationships behind compositional changes can be clearly defined. This causality-based approach could empower the nanomedicine community to better anticipate how shifts in the physicochemical properties of nanoparticles affect the protein corona composition. For instance, this strategy offers a high-throughput potential for predicting the biological identity of various nanoparticle formulations intended for safe and effective genomic medicine delivery systems. This is particularly valuable given the challenges associated with in vivo evaluations and comparisons, which can be prohibitively expensive and time-consuming due to large-scale animal studies.^18−22^ By understanding the actual causality linked to the essential components of nanoparticles, such as their composition, physicochemical properties, and surface coatings, we can better predict the biological fate and pharmacokinetics of nanomedicines. This will facilitate high-throughput screening and enable precise analysis of their biomolecular corona.^22^

## Actual Causality

While there are multiple interpretations of the concept of causality,^23,24^ we focus here on the definition of *actual causality* as articulated by Halpern and Pearl.^7^

This definition primarily addresses what Halpern and Pearl term *token-level causality*, which examines the causal effect of specific individual events. In contrast, *type-level causality* (e.g., Pearl’s Causality^24^), involves generalized causal statements, such as “smoking causes cancer”. Additionally, Ganger causality^23^ is employed to analyze time series data, enabling the prediction of one time series based on observations from another; however, it primarily offers predictive insights rather than explicit causal interpretations.

Actual causality is based on *counterfactual reasoning*, which involves considering not only what actually occurred but also what might have happened under different circumstances. This type of reasoning, often encapsulated in questions such as “What might have happened if...?”, enhances our ability to imagine alternate scenarios (counterfactual worlds) as opposed to our current reality, which is referred to as the *actual world*. By imagining different outcomes based on varied actions or events, we build our capacity to understand and analyze the implications of those hypothetical changes.

Counterfactual reasoning can be ensured through a *contingency* mechanism, in which all events are kept identical to those in the actual world, except for altering the cause of its counterfactual setting. This approach effectively isolates the true cause. Counterfactual reasoning provides Halpern and Pearl’s actual causality framework with a significant advantage, as it offers a mathematical foundation to rigorously establish the causal effects of specific events within a system. In contrast, frameworks such as type-level causality typically yield only general statements about scientific or natural phenomena, lacking precise causal specificity.

**Figure 2:**
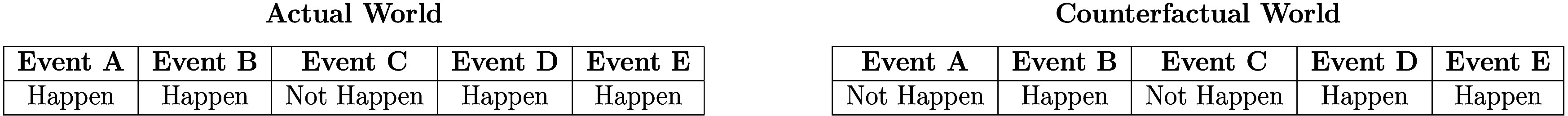
Actual and counterfactual world example.

Example 1: *To illustrate the concept of counterfactual reasoning, consider the five events shown in*Figure 2. *The left side of the figure represents the actual world. The counterfactual setting for Event A, shown on the right side, depicts a scenario in which Event A does not happen, while all other events remain as they are in the actual world. This contingency ensures that the setting is identical to the actual world for all events except Event A, thereby allowing us to isolate and examine the causal impact of Event A.*

In many studies, including protein corona reports,^1−5^ statistical methods such as artificial intelligence (AI) and machine learning are being used to analyze experimental findings, often focusing on the correlation between events. This leads to the concept of correlation versus causation. In correlation analysis, two events that occur together may appear related, moving in the same direction either positively or negatively. However, this does not imply causation, as a third *confounding factor* can influence both events, creating the illusion of a relationship. For example, as discussed earlier, “summer” or “hot weather” can increase both ice cream sales and shark attacks without one causing the other. Machine learning models often face this issue, as they are typically designed to capture correlations rather than true causal relationships. In contrast, actual causality relies on counterfactual reasoning, which allows us to identify real causal links between events rather than coincidental correlations.

Definition 1: A *basic causal model denoted as*, *consists of variables and functions that map values to these variables. The notation X* ← *x indicates that the variable X is assigned the value x. Likewise, X⃗←x⃗ denotes a vector of values x⃗ to be assigned to a vector of variables X⃗.*

Example 2: *Considering the causal model derived from the data sets obtained from experiments conducted in the report,*(16)*we denote this causal model as*. *In*, *we have four features: Protein_ID, Small_Molecule, Concentration_of_Molecule, and log2FC (Log2 fold change). The variables in our causal model correspond to these features, and the function maps the domain (values obtained from the samples) to these variables. For example, the variable Concentration_of_Molecule can take on three values: 10, 100, and 1000 μg/mL. When we write Concentration_of_Molecule ← 10, we assign the value 10 to the variable Concentration_of_Molecule.*

Definition 2: *X⃗ ← x⃗ is an actual cause of effect φ in causal setting*, *if the following three conditions hold:*

- ***AC1.** Both X⃗ ← x⃗ and φ happen.*
- ***AC2.** In the counterfactual world where we intervene on X ( X ← x′) in the actual world, the effect will not occur under the same contingency (the same setting as in AC1).*
- ***AC3.** The vector assignment X⃗ ← x⃗ is minimal; no subset of the value assignments in X⃗ ← x⃗ satisfies AC1 and AC2.*

Example 3: *In our running example (Example 2), the effect is measured by Log2 Fold Change (Log2FC). For highly abundant proteins, the threshold is set at Log2FC ≥ 0.1, whereas for other proteins, it is set at Log2FC ≤ 0.1. For instance, in our data set, if a sample is listed among the highly abundant proteins and the Log2FC value is 2, the effect φ happens. Conversely, if a sample is not listed among the highly abundant proteins and the Log2FC value is 2, the effect does not happen.*

The selection of this threshold for highly abundant proteins aims to identify which small molecules can further decrease the abundance of albumin in the protein corona profile. The term Log2FC refers to the log base 2 fold change of “protein abundance in small molecule-treated protein corona” relative to “protein abundance in untreated protein corona.”

It is noteworthy that, for highly abundant proteins, a Log2FC ≥ 0.1 indicates that albumin and other highly abundant proteins, defined as the top 25 most abundant proteins in plasma when analyzed without nanoparticles via mass spectrometry, become enriched in the protein corona following small molecule treatment. This suggests that small molecules with a Log2FC ≥ 0.1 are not suitable for our purposes, as an increase in highly abundant proteins may decrease the depth of proteome coverage. Conversely, for our search for small molecules that can enhance the depth of human plasma proteome coverage, we focus on highly abundant proteins with a Log2FC < 0.1. For low-abundance proteins, the opposite is true: we seek small molecules that yield a Log2FC > 0.1, indicating an enrichment of these proteins in the protein corona.

In this study, we are considering the *probabilistic* actual causation, which is the extension of Halpern and Pearl’s definition from Fenton-Glynn.^25,26^ Fenton-Glynn extended Halpern and Pearl’s definition into a probabilistic version by adhering to the probability-raising principle, which is a traditional approach in the study of probabilistic causation. The core idea of this principle is that a cause should increase the probability of its effect. In this definition, we are changing **AC2** to a probabilistic version, called **PC2**. In this new definition, we state that the probability of the actual world occurring is greater than that of the counterfactual world. Specifically, the probability of the actual world occurring (where cause *X* ← *x* and effect φ happen together) is greater than the probability of the counterfactual world occurring (where we intervene on *X* ← *x*′ and effect φ happens) under the same conditions.

## Algorithmic Search for Actual Causality

Given a data set of variables along with their values, we begin by searching through samples where the effect occurs, ensuring that **AC1** is satisfied. Within these samples, we calculate the probability of the effect occurring in both the actual world and a counterfactual world (where the feature’s valuation is altered). If the probability of the effect occurring in the actual world is higher than that in the counterfactual world, the feature’s valuation satisfies **PC2**. In this study, we are seeking a singleton cause (a cause represented by a single variable), which means that it is already in its minimal form and satisfies **AC3**. Consequently, we identify the actual cause for this subproblem. We have implemented this algorithm in the Python programming language.^27^ We assume that the causal analysis is based on the data set available to us; therefore, the identified causal relationships may change if the data set is modified.

## Results and Discussion

Liquid chromatography mass spectrometry was used to analyze the protein corona compositions of various protein coronas formed after interactions between polystyrene nanoparticles and both untreated and small molecule-treated plasmas; full experimental details and data analysis is available in our recent report.^16^ It is noteworthy that the addition of small molecules to plasma proteins can change the interaction sites of proteins with nanoparticles and, therefore, manipulate protein corona composition. Small molecules enhanced the detection of proteins by reducing the attachment of highly abundant plasma proteins to the nanoparticle surface while simultaneously increasing the attachment of low-abundance proteins. Cumulatively, we detected 1793 proteins with the array of small molecules, compared to 681 proteins in untreated plasma.^16^ For example, molecular dynamics analysis of the interactions between PtdChos and albumin revealed the presence of hydrophobic interactions, hydrogen bonds, and water bridges.^16^ These interactions can decrease the affinity of albumin for the surface of hydrophobic polystyrene nanoparticles, thereby facilitating the attachment of low-abundance proteins to the nanoparticle surfaces. As each of the small molecules could increase the numbers of detected proteins in a protein corona, we were curious to identify the main cause for quantifying such a high number of the proteins. From the experimental outcome, we could only observe that PtdChos exerted a substantial increase in protein detection at the concentration of 1000 μg/mL. To apply this concept to causality, we calculated the abundance ratio of each protein in the corona of untreated plasma to that of treated plasma and presented their log2FC values (see Supplementary Data 1 for details; explanations on the treatment of the missing values for some small molecules are provided in the original research^16^) for our causality analysis, alongside three other features: Protein_ID, Small_Molecule, and Concentration_of_Molecule. A list of descriptions for the variables can be found in Table 1. In the causal model, these features serve as variables, with the effect measured by Log2FC (Log2 fold change), which has a threshold of 0.1 or more for highly abundant proteins (as listed in Table. 2) and 0.1 (or less) for the rest of the identified proteins in the protein corona, highlighting the need for depletion of highly abundant proteins and enrichment of the low abundance proteins. It is noteworthy that the top 25 highly abundant proteins were selected based on their relative abundance (mean normalized intensities across three replicates), in a pooled plasma sample analyzed by LC-MS in our previous study.^16^

**Table 1:** Description of Variables Causal Model

Variable	Description
Protein_ID	Protein accession numbers are unique identifier of each of the detected protein sequences.
Small_Molecule	The added vitamins, lipids, nutrition, and metabolites to plasma that created various protein corona profiles.
Concentration_of_Molecule	The concentration of each small molecule added to the plasma: 10, 100, and 1000 μg/mL
log2FC	Log2 fold change of (protein abundance in small molecule-treated protein corona)/(protein abundance in untreated protein corona)

**Table 2:** List of Highly Abundant Proteins

Index	Protein ID	Protein Name
1	P02768	Albumin
2	P02787	Serotransferrin
3	P00738	Haptoglobin
4	P01876-1	Isoform 1 of Immunoglobulin heavy constant alpha 1
5	P02671	Fibrinogen alpha chain
6	P02675	Fibrinogen beta chain
7	P01024	Complement C3
8	P02679	Fibrinogen gamma chain
9	P02647	Apolipoprotein A-I
10	P02790	Hemopexin
11	P02652	Apolipoprotein A-II
12	P02774	Vitamin D-binding protein
13	P01023	Alpha-2-macroglobulin
14	P01860	Immunoglobulin heavy constant gamma 3
15	P02765	Alpha-2-HS-glycoprotein
16	P08603	Complement factor H
17	P01871-1	Isoform 1 of Immunoglobulin heavy constant mu
18	P00450	Ceruloplasmin
19	P00751	Complement factor B
20	P01857-1	Isoform 1 of Immunoglobulin heavy constant gamma 1
21	P01042-2	Isoform LMW of Kininogen-1
22	P0DOX7	Immunoglobulin kappa light chain
23	P02763	Alpha-1-acid glycoprotein 1
24	P00747	Plasminogen
25	P04217	Alpha-1B-glycoprotein

Our objective in this approach was to identify the actual causes by examining various threshold combinations for highly abundant proteins. We divided the spectrum into two segments: thresholds between the minimum value of Log2FC and 0.1 for highly abundant proteins and thresholds between 0.1 and the maximum value of Log2FC for the remaining proteins. At each step, we establish two thresholds, one for highly abundant proteins and one for the remaining ones. The effects were determined using these thresholds: if the Log2FC value was less than or equal to the threshold set for highly abundant proteins or greater than the threshold set for the remaining proteins, the effect was considered to have occurred; otherwise, it was considered absent. Following this determination, we then sought to identify the cause of these observed effects.

**Figure 3:**
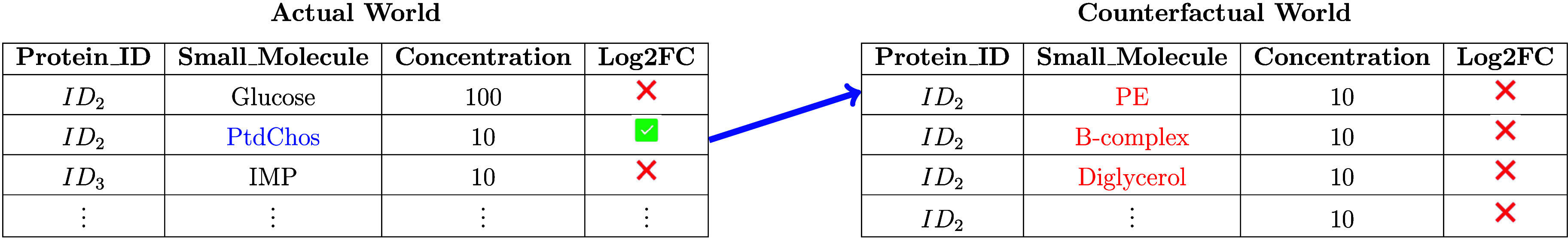
Procedure of counterfactual reasoning. The left panel illustrates the actual world, and the right panel depicts the counterfactual world.

In Figure 3, the procedure for finding causation by using counterfactual reasoning is illustrated. The left table represents the actual world, where we search for samples (values assigned to variables) in which the effect has occurred. The right table represents the counterfactual world, where we search for samples that differ only in the variable of interest, while keeping the other variables the same. We then calculate the probability of the effect occurring in the actual world compared to the counterfactual world. If the probability in the actual world exceeds that in the counterfactual world, the variable’s assigned value can be identified as the cause (for instance, in Figure 3, the cause is *Small*_*Molecule* ← *PtdChos*). Next, we calculated the probability of the effect occurring in the actual world. Specifically, we determined how often the cause and effect occur together and divide this by the total number of occurrences of the cause event. Importantly, this probability does not serve to directly establish the causation. Instead, causation is identified using the algorithm provided, after which we calculate the probability of the effect’s occurrence, conditioned on the presence of the identified cause.

**Figure 4:**
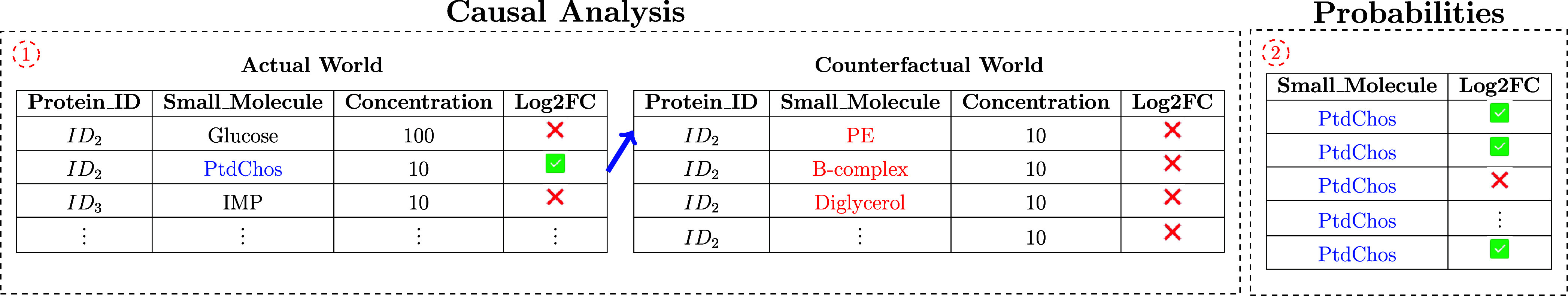
Example of how cause can be computed and how to determine what the probability of it is.

Example 4: *In our ongoing Example 3, after identifying the effect using the high and low thresholds established for Log2FC, we proceed to determine its cause. In Figure 4, we start with causal analysis by identifying Small_ Molecule ← PtdChos as a potential cause, where the effect is also observed, thus satisfying **AC1** (the actual world). We then verify **PC2** through counterfactual reasoning, examining all counterfactual worlds where Small_ Molecule ←̷ PtdChos, meaning Small_ Molecule can take any value except PtdChos while other variables (Protein_ ID and Concentration_ of_Molecule) remain constant. In these scenarios, we compare the probability of the effect occurring in the actual world against its probability in the counterfactual worlds; if the probability in the actual world is higher, then we identify the cause. This approach may yield multiple potential causes; therefore, we then focus on identifying the most probable ones. The second step involves calculating conditional probabilities to assess the likelihood of the effect for each identified cause. For instance, if Small_ Molecule ← PtdChos is identified as a cause, we check all instances where Small_ Molecule ← PtdChos occurs and count how often the effect is also observed, calculating the probability by dividing the instances where both cause and effect co-occur by the total instances of Small_ Molecule ← PtdChos. This gives us the conditional probability of the effect given the cause. By calculating these probabilities, we identify and return the top three causes with the highest likelihood, highlighting the most probable causes.*

**Figure 5:**
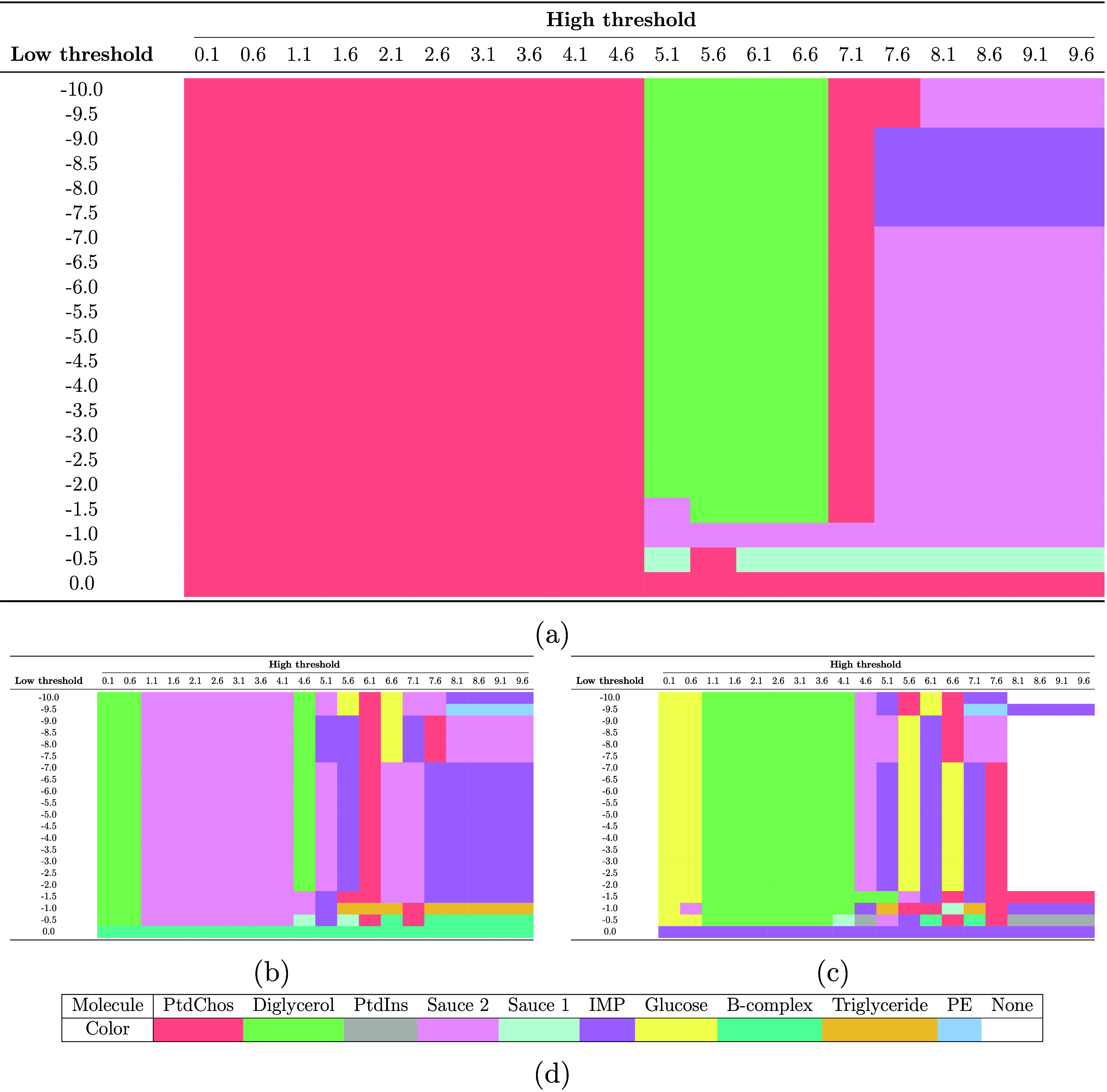
Actual cause with the (a) highest, (b) second highest, (c) and third highest probabilities in different threshold settings. Details about the colors of each cell can also be found in panel d. Employed molecules in this study are glucose, triglyceride, diglycerol, phosphatidylcholine (PtdChos), phosphatidylethanolamine (PE), l-α-phosphatidylinositol (PtdIns), inosine 5-monophosphate (IMP), and vitamin B-complex. Sauce 1 and Sauce 2 are combinations of four small molecules each. Molecular Sauce 1 contains glucose, triglyceride, diglycerol, and PtdChos, while Molecular Sauce 2 consists of PE, PtdIns, IMP, and vitamin B-complex. Additionally, “None” indicates that no cause with any probability was found for that cell with the given threshold setting. We set the threshold for low-abundance proteins from 0.1 to the maximum observed value in the data set and that for highly abundant proteins from 0.1 to the minimum observed value.

In Figure 5, we determined the actual cause with the highest, second highest, and third highest probabilities by applying our mathematical approach. This method employs a low threshold for highly abundant proteins and a high threshold for the low abundance proteins. In each cell shown in Figure 5, the causes with the highest, second highest, and third highest probabilities are identified (Figure 5a,b,c). Our results revealed that more than 50% of the causes with the highest probability are related to the spiking of PtdChos (See Table. 3 for more details). Further analysis revealed that in over 65% of the settings with different thresholds, PtdChos is among the top three causes with the highest probability. This outcome is particularly intriguing, as the experimental correlation data indicated the critical role of PtdChos only at the highest concentration used (i.e., 1000 μg/mL). In contrast, the causality results demonstrated the critical role of PtdChos independent of its concentration. From the biological point of view, the mechanistic role of PtdChos in enhancing the depth of the plasma proteome lies in its interaction with the hydrophobic sites of albumin.^16,28,29^ This interaction reduces albumin’s affinity for the surface of hydrophobic polystyrene nanoparticles. Consequently, this allows for the increased enrichment of low-abundance proteins on the nanoparticle surface, thereby expanding the proteome coverage. We have validated this hypothesis using a molecular dynamics simulation approach, as detailed elsewhere.^16^ We also anticipate that PtdChos will significantly enhance the depth of the plasma proteome primarily in hydrophobic nanoparticles due to its interaction with the hydrophobic sites of albumin.

**Table 3:** Occurrences of Molecules from Figure 5a–c under Different Threshold Conditions

Molecule	PtdChos	Sauce 2	Diglycerol	IMP	others
1st Prob Cause Frequencies (5a)	57.38%	18.81%	16.9%	4.76%	2.15%
2nd Prob Cause Frequencies (5b)	6.19%	47.38%	13.57%	21.42%	11.43%
3rd Prob Cause Frequencies (5c)	7.14%	7.61%	33.57%	17.38%	34.3%

In our study, we seek to identify causal effects that satisfy **AC1**, where both the cause and effect occur, and **PC2**, involving counterfactual reasoning, which states that the probability of the effect occurring in the actual world is greater than that in the counterfactual world. If an event meets these criteria, we can claim a causal relationship between two events. **AC3** is inherently satisfied in our analysis, as we consider only singleton causes. Thus, our causal analysis relies heavily on counterfactual reasoning, requiring us to consider all alternative scenarios that differ in only one variable’s value while keeping others constant. However, it is impractical to account for all possible scenarios, as doing so would require an infinite number of samples. Consequently, the size of our data set becomes a limiting factor; a larger data set enhances our confidence that our findings more accurately reflect real-world causality. Ultimately, the causal conclusions drawn here are specifically derived from our data set, distinguishing our approach from type-level causality, which provides general rather than event-specific causal statements.

In summary, we have introduced the concept of actual causality and demonstrated its critical importance over statistical correlation in mechanistically understanding the causes of compositional changes in the protein corona. Specifically, we examined the intricate dynamics of the protein corona, focusing on the role of small-molecule-spiked plasma in enhancing nanoparticle efficacy in proteomics. By employing a causality-based approach, we moved beyond traditional correlation methods to rigorously define the actual causes of these compositional changes. Our findings revealed the significant impact of PtdChos in the protein corona, highlighting its critical role independent of concentration levels. This actual causality analysis is not a prediction problem like those typically addressed in traditional AI and machine learning; however, it offers valuable prior knowledge on how specific small molecules influence nanoparticle interactions with biological systems, which can, in turn, support more accurate predictions in future analyses. In addition, it provides a unique opportunity to look into the derivatives of small molecules of actual cause to even more improve the deep proteomic profiling of plasma. This causality-based perspective in the field of protein coronas offers a high-throughput predictive capability, enabling more precise design with predictable protein corona nanoparticles tailored for therapeutic and diagnostic purposes. As we continue to refine our understanding of the protein corona, the insights gained from this study pave the way for safer and more effective nanomedicine applications, ultimately contributing to the advancement of personalized medicine.

## Data Availability

The developed code and related data are available through the following link: https://github.com/arshiarafiei/Causality_Analysis_of_Protein_Corona. The mass spectrometry data for all bottom-up experiments have been deposited in the MassIVE database under the identifier MSV000094257. Additionally, the MS RAW files for the top-down proteomics analysis have been submitted to the ProteomeXchange Consortium via PRIDE under data set identifier PXD053359.

## Supporting Information Available

Details of the mass spectrometry outcomes (with permission from ref (16)); *P*-values were calculated using a two-sided Welch’s *t* test, based on the normalized protein intensities in each sample compared to the untreated corona within replicates (XLSX)
Statistical analysis of the causality of the top three small molecules in each of the predetermined thresholds (XLSX)
